# Multiple High Grade Rhabdoid Papillary Meningiomas Mimicking Choroid Plexus Carcinoma: A Case Report

**DOI:** 10.30699/ijp.2019.80193.1757

**Published:** 2019-09-22

**Authors:** Arezoo Eftekhar-Javadi, Dorna Motevalli, Ahmad Pourrashidi Boshrabadi, Hedieh Moradi-Tabriz, Hoda Asefi

**Affiliations:** 1 *Department of Pathology, Sina Hospital, Tehran University of Medical Sciences, Tehran, Iran*; 2 *Department of Neurosurgery, Sina Hospital, Tehran University of Medical Sciences, Tehran, Iran*; 3 *Department of Radiology, Sina Hospital, Tehran University of Medical Sciences, Tehran, Iran*

**Keywords:** Meningioma (M0013362), Malignant meningioma (M0337826), Papillary meningioma (M0337837)

## Abstract

Rhabdoid papillary meningioma is an uncommon aggressive variant of meningioma which has the potential to metastasize and spread throughout the brain and even out of the cranium. Herein, we present recurrence of the brain tumor in a 26-year-old woman. The patient had history of the surgery for two lesions in the right temporal lobe and the left cerebellopontine angle. Imaging showed three lesions in the right temporal lobe, the right occipital horn wall, and the left cerebellopontine angle. These radiologic findings were mostly suggestive of atypical meningioma. In the surgical view, the mass was solid-cystic reddish Cauliflower-shaped in the right temporal lobe attaching to the temporal horn. The microscopic examination showed a cellular neoplasm with the sheet-like and papillary growth pattern. Individual cells had vesicular nuclei some with prominent nucleoli and eosinophilic cytoplasm. The areas of the tumor cells showed round eccentric nuclei and prominent nucleoli with eosinophilic cytoplasm. Immunohistochemistry studies showed diffuse positivity of tumor cells with Vimentin, EMA, and S100. The overall clinical, radiological and histopathological examinations were compatible with high grade rhabdoid-papillary meningiomas. In the present case study, we discuss imaging and histomorphological features of this rare entity of meningiomas.

## Introduction

Meningiomas are a group of tumors with menin-gothelial origin and wide spectrum of the histological appearances. Rhabdoid meningioma was first described in 15 cases in 1998 and then categorized as grade III meningioma in 2000 (1, 2). Malignant meniongiomas including Papillary meningioma, Rhabdoid meningioma and Anaplastic meningioma account for less than 5 percent of all meningiomas. These tumors behave more aggressive and are categorized as grade III according to the world health organization (WHO) classification and have a tendency to invade brain, bone tissue, extracranial tissue as well as metastasis (3). An important feature of papillary meningioma is perivascular pseudorosettes which mimics ependymoma and choroid plexus neoplasms. Rhabdoid meningioma consists of tumor cells that resemble rhabdomyoblasts without having skeletal muscle differentiation and show the nuclear pseudoinclusion and grooves (4, 5). Meningiomas with papillary and partly rhabdoid features have been reported rarely in the literature, and can be misdiagnosed as ependymoma, or choroid plexus carcinoma, therefore, pathologists should be aware of this tumor entity (1,6). Herein, we present a rare case of rhabdoid papillary meningioma recurrence with the discussion of clinical, imaging and histopathologic examination.

## Case Report

The case was a 26-year-old woman who had surgery previously for two lesions in the right temporal lobe and the left cerebellopontine angle. She had a previous history of brain masses which was diagnosed as ependymoma and choroid plexus carcinoma according to the pathology reports. In follow-up period, her evaluations showed new lesions in the brain. Imaging showed three lesions in the right temporal lobe, the right occipital horn wall, and the left cerebellopontine angle which were consistent with multiple meningiomas. 

**Radiological and Surgical Evaluation**

**Fig. 1:**
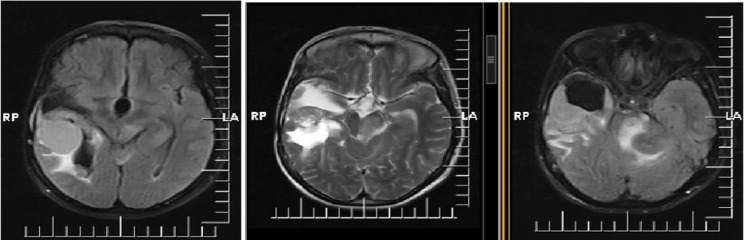
Lesions in the right temporal lobe, the right occipital horn wall, and the left cerebellopontine angle

The images showed three lesions in the right temporal lobe, the right occipital horn wall, and the left cerebellopontine angle (Figure 1). There is a 67*30*39 mm low enhancing mass with heterogenous signal intensity in T2 weighted images and cystic component in the right temporal region. It is mostly extra-axial and has dural tail but has invaded right temporal lobe and abuts right lateral ventricle. The findings are mostly suggestive of atypical meningioma. An enhancing 10*13 mm low T1/T2 signal intensity mass is seen in occipital horn of the right lateral ventricle. Another 35*30*40 mm low T1/heterogenous T2 signal intensity mass with the cystic component and heterogenous enhancement are seen in the left cerebellopontine angle with no obvious extension or widening left internal acoustic canal. It has compressive effect and edema in the brain stem, left cerebellar hemisphere and left middle cerebellar peduncle. These two masses are also mostly suggestive of meningioma. Acoustic schwannoma is the differential diagnosis of cerebellopontine mass. 

In the surgical view, mass was solid-cystic reddish Cauliflower-shaped in the right temporal lobe attached to the temporal horn. 

**Fig. 2:**
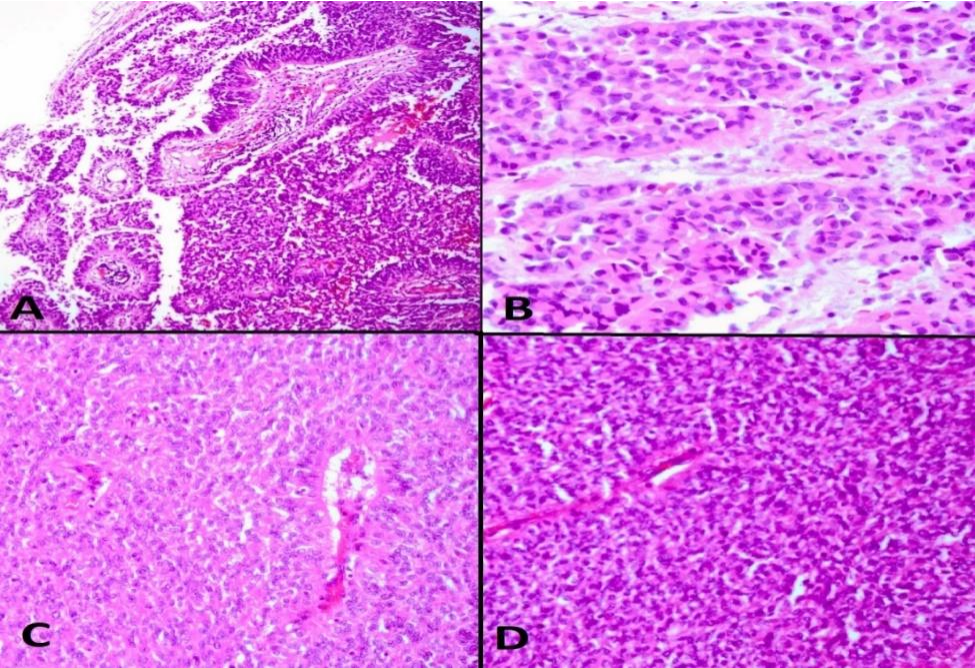
Histomorphological features of the tumor. A) Papillary and perivascular pseudo-rosette like areas (H&E staining, 100X), B) Rhabdoid cells (H&E staining, 400X), C, D) Sheet-like tumoral areas (H&E staining, 200X)

Microscopically, it showed a well-circumscribed cellular neoplasm with sheet-like growth and pseu-dopapillary pattern. Individual cells have vesicular nuclei some with prominent nucleoli and eosinophilic cytoplasm. The tumor cells are frequently arranged around the blood vessels (perivascular pseudo-rosette like). The areas of tumor cells had round eccentric nuclei and prominent nucleoli with eosinophilic cytoplasm with occasional intracytoplasmic hyaline inclusions (rhabdoid cells). The mitotic figures were also noted (Figure 2).

**Immonohistochemistry**

**Fig. 3:**
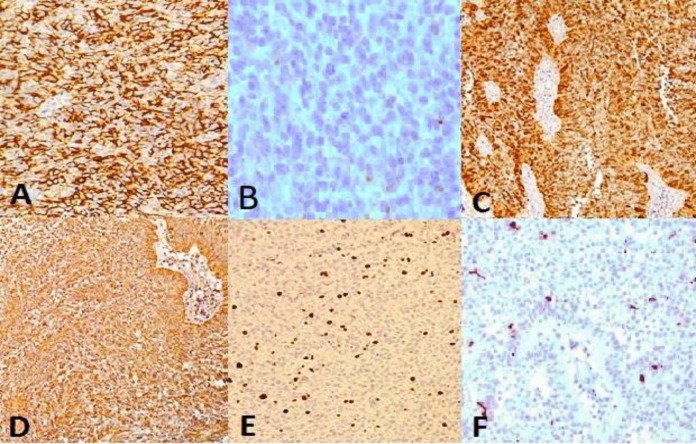
Immunohistochemistry staining for A) Diffuse and intense staining of EMA, B) Negative PR, C) Positive S100 immunostaining, D) Diffuse and intense staining of Vimentin, E) Ki67 shows at least 10% proliferative index, F) Negative GFAP

Tumor cells were highlighted diffusely and intense with EMA, vimentin and S100 marker. The negativity of GFAP, Myogenin, Desmin, Synaptophysin and PR was also noted. The Ki67 showed at least 10% proliferative activity in tumor cells (Figure 3).

## Discussion

Herein, we present an unusual case of high grade meningioma with papillary rhabdoid features in a 26-year-old female patient. The main diagnostic challenge was differentiation between ependymoma, choroid plexus carcinoma and papillary-rhabdoid variant of meningioma. Occasional pseudorosettes were reminiscence of ependymoma and the tumor was previously diagnosed as ependymoma and choroid plexus carcinoma in main and review pathology reports of primary mass surgical excision. Brain MRI revealed a 67*30*39 mm low enhancing mass with heterogenous signal intensity in T2 weighted images and cystic component in the right temporal region which was mostly extra-axial with dural tail but has invaded right temporal lobe and abuts right lateral ventricle. The findings were mostly suggestive of atypical meningioma. An enhancing 10*13 mm low T1/T2 signal intensity mass was seen in the occipital horn of the right lateral ventricle. Another 35*30*40 mm low T1/heterogenous T2 signal intensity mass with cystic components and heterogenous enhancement was also seen in the left cerebellopontine angle with no obvious extension or widening left internal acoustic canal. There was also compressive effect and edema in brain stem, left cerebellar hemisphere and left middle cerebellar peduncle. These two masses were also mostly suggestive of meningioma but acoustic schwannoma was the differential diagnosis of cerebellopontine mass. Therefore, radiologist was suspicious about the neurofibromatosis type II which was ruled out by neurologists. 

The recurrent mass which was sent to our department was evaluated histomorphologically. Microscopically, the tumor was composed of two patterns of papillary and rhabdoid sheets of tumoral cells. Immunohistochemistry showed strong and diffuse immunoreactivity of tumor cells to vimentin and EMA which was in favor of meningiomatous nature of the tumor and ruled out ependomoa. The S100 was also positive. GFAP, cytokeratin, synaptophysin and PR were negative. The negativity of tumor cells for CK ruled out the metastatic nature of the lesion. These findings established the diagnosis of malignant meningioma. 

Grade III meningiomas are currently categorized on the basis of their architectural pattern into three subclasses of papillary, rhabdoid and anaplastic (3, 7). However, some meningiomas have a combination of the morphological features. A rare variant is rhabdoid papillary meningioma which has a combination of papillary architecture and rhabdoid cytomorphology (6). To our knowledge less than 30 cases have been reported in the English language literature. All the cases had an aggressive course. The local recurrences, spreading through CSF and even metastasis are common in this variant of meningioma (3, 8-12).

The largest case series was reported by Wu *et al.*, in which nine specimens from 6 patients (3 male and 3 female) were evaluated. In this study, the mean age of the patients was 44.7. Five cases had episodes of the recurrence and one patient died due to diffuse leptomeningeal dissemination. One of the patients had no tumoral recurrence after 8 years follow-up and in this study all of the tumors were both EMA and vimentin positive. The Ki67 index (MIB-1) was higher in tumor recurrences (3).

In a search with keyword “papillary rhabdoid meningioma” in PubMed, the first case was reported by Hojo *et al*. which was a 15 year-old boy with a mass in posterior fossa with extra-cranial extension during 6 years. In this case, regional and histomorphological resemblance (perivascular and pseudopapillary growth) of tumor to ependymoma and also immunohistochemical positivity of some tumoral cells with GFAP, Neurofilament and alpha smooth muscle actin caused diagnostic challenge but positive EMA and vimentin and electron microscopic examination helped to establish the diagnosis of papillary rhabdoid meningioma (6). Unusual expression of GFAP was also reported in other highly aggressive case by Eom *et al*. (13).

Another case reported by Al-Habib *et al*. was a 27 year-old female patient with highly anaplastic rhabdoid papillary intracerebral meningioma with leptomeningeal spread and CSF metastasis to the cervical cord. Immunohistochemical examination of the tumoral cells showed positivity of EMA, vimentin and S100 and Ki67 index of 80%. This case shared positivity of S100 with our case although our case had higher proliferative index in comparison (80% vs 10%) (14).

According to the histopathological findings, this unusual variant of meningioma, may cause diagnostic challenge and must be differentiated from ependymoma, choroid plexus carcinoma and metastatic carcinomas. Our patient died few months after the surgery. Rhabdoid papillary meningiomas are highly aggressive, thus, close follow-up of the patient after the surgery is mandatory.
